# Impact of *Aspergillus fumigatus *in allergic airway diseases

**DOI:** 10.1186/2045-7022-1-4

**Published:** 2011-06-10

**Authors:** Neelkamal Chaudhary, Kieren A Marr

**Affiliations:** 1 School of Medicine, Johns Hopkins University, Baltimore, MD, USA; 2 The Sidney Kimmel Cancer Center, Baltimore, MD, USA

## Abstract

For decades, fungi have been recognized as associated with asthma and other reactive airway diseases. In contrast to type I-mediated allergies caused by pollen, fungi cause a large number of allergic diseases such as allergic bronchopulmonary mycoses, rhinitis, allergic sinusitis and hypersensitivity pneumonitis. Amongst the fungi, *Aspergillus fumigatus *is the most prevalent cause of severe pulmonary allergic disease, including allergic bronchopulmonary aspergillosis (ABPA), known to be associated with chronic lung injury and deterioration in pulmonary function in people with chronic asthma and cystic fibrosis (CF). The goal of this review is to discuss new understandings of host-pathogen interactions in the genesis of allergic airway diseases caused by *A. fumigatus*. Host and pathogen related factors that participate in triggering the inflammatory cycle leading to pulmonary exacerbations in ABPA are discussed.

## Review

### Fungi and Respiratory Allergy

Fungi are ubiquitous and responsible for causing a broad spectrum of type I-IV hypersensitivity diseases [1]. Recent epidemiologic studies clearly outline the link between fungal sensitization and exacerbations of allergic asthma, leading to increased morbidity and mortality [2-4]. The major respiratory manifestations caused by fungi include allergic bronchopulmonary mycoses (ABPM), severe asthma with fungal sensitization (SAFS), hypersensitivity pneumonitis, fungal sinusitis and allergic rhinitis [1]. In contrast to other allergens (e.g. pollen), fungi also pose a life-threatening risk for invasive pneumonia in immunocompromised patients; further emphasizing their significant impact on human health. It is now understood that the pathogenesis of diseases like asthma and allergy is determined by the interactions between host, genes and environment [5,6]. In this review, we focus on the role of filamentous fungi in respiratory allergic diseases, and discuss how fungi mediate T helper (Th) 2 -mediated allergic diseases as a result of host-pathogen interactions that lead in ineffective clearance of spores, and how predisposing factors like host genetics determine outcomes for respiratory diseases.

### Epidemiology and Outcomes

Amongst the filamentous fungi, *Aspergillus *species have been strongly linked with exacerbations of asthma and other respiratory allergic diseases [2,7]. Over 80% of *Aspergillus*-related conditions, such as extrinsic allergic alveolitis, asthma, allergic sinusitis, chronic eosinophilic pneumonia, hypersensitivity pneumonitis, SAFS, and allergic bronchopulmonary aspergillosis (ABPA) are most frequently caused by *A. fumigatus *[8]. ABPA is the most complex allergic manifestation caused by *A. fumigatus*, and was first reported in the United Kingdom by Hinson *et al. *in 1952 [9]. Other fungi such as *Cryptococcus neoformans *and *Scedosporium apiospermum *are also associated with similar clinical manifestations broadly referred to as ABPM.

Improved diagnostic methods and awareness have led to recent reports of higher prevalence of ABPA in patients suffering from chronic asthma (1-40%) and acute severe asthma (~38%) [10-12]. The prevalence of *A. fumigatus *hypersensitivity is even higher in patients with acute severe asthma (~51%) [12]. *A. fumigatus*-sensitized asthmatic patients have been reported to have poorer lung function [13,14]. ABPA is also prevalent in up to 7-15% of cystic fibrosis (CF) patients [15-17]. ABPA leads to poorly controlled asthma with pulmonary exacerbations and detrimental consequences; dependence on oral-corticosteroids increases the risk for secondary infections [18]. In rare cases, ABPA disease has also been reported to complicate other lung diseases including idiopathic bronchiectasis, chronic obstructive pulmonary disease (COPD) and chronic granulomatous disease [19-21]. Moreover, ABPA has also been reported in patients with pulmonary aspergilloma and chronic necrotizing pulmonary aspergillosis (reviewed in [16]). Diagnostic parameters of ABPA include asthma, roentgenographic fleeting pulmonary opacities, central bronchiectasis, type I and type III hypersensitivity to *A. fumigatus *antigens (discussed in more depth below), and increased peripheral blood eosinophilia [22]. ABPA includes several stages of exacerbations (acute and recurrent) and remissions, central bronchiectasis with pulmonary fibrosis and a possible respiratory failure [22]. However, not all ABPA patients at different stages develop these diagnostic criteria, and some of these features overlap with those of *A. fumigatus *hypersensitivity and asthmatic patients. Uniformity of diagnostic parameters is still needed for improving outcomes in ABPA patients.

### Pathogenesis of Fungi & Airway Clearance

The most common predisposing factor associated with ABPA pathogenesis is defective clearance of conidia in airways. Airway epithelium, as the first line of defense, extrudes inhaled fungal spores through mucociliary action. Fungal spores evading epithelial mucociliary clearance reach alveoli, and are dealt with by resident phagocytes; neutrophils, as effector cells, efficiently kill germinated hyphal forms through non-oxidative or oxidative-mediated responses. Airway myeloid cells also recognize fungi through pattern recognition receptors (PRRs) such as toll like receptors (TLRs) and Dectin-1, and stimulate the secretion of proinflammatory cytokines/chemokines [8,23]. A breached innate immune defense by fungal spores is required for their germination and establishment of fungal-mediated allergies as dormant conidia are immunologically inert [24]. It is thought that ineffective clearance of spores results largely from structural abnormalities in the airway epithelium, as observed in patients with allergic asthma or other causes of chronic lung disease, allowing for germination of spores into vegetative cells (hyphae) [4,25-29].

Fungal hyphae secrete proteases and toxins that damage the airway epithelium, leading to the loss of tight junctions. Epithelium damage leads to increased exposure of *A. fumigatus *antigens to pulmonary dendritic cells (DCs), which prime naïve Th-cells to Th2 that secrete cytokines such as IL-4, IL-5 and IL-13 leading to IgE isotype switching of B-cells, increased secretion of *A. fumigatus*-specific IgG, IgE and total IgE antibodies, and pulmonary eosinophilic influx. More than 20 allergens/antigens of *A. fumigatus *have been described to date (http://www.allergen.org). Moreover, several chemokines such as Monocyte Chemotactic protein (MCP-1), Regulated on Activation and Normal T-cell expressed (RANTES), IL-8 and macrophage inflammatory protein-1α (MIP-1α) secreted by phagocytic and non-phagocytic cells, perpetuate inflammatory pathology of ABPA [17].

### Immunopathogenesis of ABPA in CF patients: Newer Understandings

CF is caused by mutations in cystic fibrosis transmembrane conductance regulator (CFTR), present on the apical membranes of epithelial cells. Over 1,500 mutations in CFTR are known, and the most common is the deletion of phenylalanine at position 508 (ΔF508), which causes CFTR protein misfolding and retention in the endoplasmic reticulum (ER) [30]. Filamentous fungi are commonly isolated from sputum of CF patients and *A. fumigatus *is the most prevalent fungal species [31,32]. *A. fumigatus*-mediated chronic asthma or ABPA in CF patients significantly deteriorates lung function leading to poorer outcomes [27,32-37]. Diagnosis of ABPA in CF patients further poses a significant challenge as diagnostic criteria such as pulmonary infiltrates, bronchiectasis and obstructive lung disease are common features in CF patients with or without ABPA.

People with ABPA are known to have higher frequencies of CFTR mutations than the healthy population, suggesting that CFTR mutations possibly impact the clearance of *A. fumigatus *spores [38]. Using the bronchial epithelial cell lines and primary murine tracheal cells, we observed that CFTR mutations/deficiency impact binding and uptake of *A. fumigatus *conidia with differential secretion of inflammatory mediators by CF cells [39]. Studies have reported improved clinical outcomes for ABPA patients treated with azoles [40,41]. Moreover, anti-fungal therapy also led to the better lung function in *A. fumigatus*-sensitized CF patients [42]. These studies indicate that *A. fumigatus *actively participate in triggering Th2-type responses that perpetuates in the setting of CFTR mutations [40-42].

Several studies have linked CF genotype to cytokine dysregulation and have shown that immune responses are biased towards Th2 type with increased secretion of proinflammatory cytokines by CF epithelial cells [17,43,44]. These studies indicate that CFTR mutations lead to cytokine milieu which can shift the balance of *A. fumigatus*-specific CD4+ T-cell responses towards Th2. It is also possible that in the setting of CF, there is an increased frequency of *A. fumigatus*-specific CD4+ Th2 cells. Studies by Allard *et al. *showed that T-cells from naïve CFTR-deficient mice produce higher levels of Th2-cytokines [45]. This study also demonstrated that mice with CFTR-deficiency or mutations develop profound Th2-mediated response to hyphal antigens of *A. fumigatus *[45]. That CFTR mutations regulate Th1/Th2 balance was further evident by Muller *et al. *studies which demonstrated that intra-tracheal delivery of recombinant truncated CFTR reduces levels of Th2-cytokines and IgE antibody in CFTR-deficient mouse model of ABPA [46].

The mechanisms of Th2 bias have not been precisely defined. Besides epithelial cells, CFTR is also expressed by other immune cells such as lymphocytes, and alveolar macrophages. Di *et al. *demonstrated that CFTR-deficient alveolar macrophages fail to undergo lysosomal acidification, potentially leading to an environment conducive for the growth of pathogenic microorganisms [45]. Deficiency of CFTR on CD4+ T-lymphocytes leads to aberrant calcium fluxes causing an increased nuclear translocation of Nuclear factor of activated T-cells (NFAT) possibly driving Th2-biased responses [47]. Most recently, Kreindler *et al*. demonstrated that Th2 reactivity in CF-ABPA patients was dependent on the expression of costimulatory molecule OX40 ligand (OX40L) on DCs which decreased on *in vitro *addition of vitamin D3 [48]. Thus, CF patients exhibit multifactorial defects in both pulmonary innate and adaptive immunity to pathogens; modulation of host immunity due to the chronic airway infection with *A. fumigatus *possibly leads to the establishment of ABPA.

### Fungi- Factors and Host Immune Response

#### Fungal Proteases

Fungal proteases are potent allergens, triggering pulmonary allergic responses [7,49-51]. *Aspergillus *species are known to produce large amounts of proteases that induce IL-6, IL-8 and MCP-1 production by airway epithelial cells; these enzymes also disrupt epithelial tight junctions and induce cellular desquamation [26,52-54]. Recently, Porter *et al. *reported that proteases derived from *A. niger *induce robust allergic lung disease in mice [7].

Most recently, it has been noted that proteases activate protease activated receptors (PARs). PARs are G-protein coupled receptors present on the airway cells and other cells such as mast cells, eosinophils, neutrophils, macrophages, and lymphocytes [55]. To date, four PARs have been identified; PAR-2 is the most important in allergic airway disease owing to its increased expression on the airways of asthmatic patients [56]. Interestingly, injured airway epithelial cells also secrete trypsin, a PAR-2 agonist that further aggravates allergic inflammatory responses. Using murine models, PAR-2 has been reported to mediate pulmonary eosinophilc infiltration and airway hyperreactivity suggesting a role in exacerbating Th2-mediated responses [57]. Moreover, PAR-2 promotes fibrosis and increased IgE production in allergic diseases (reviewed in [55]). The role of TLRs in regulating PARs signaling and inflammatory responses to *A. fumigatus *has also been reported. Studies by Moretti *et al. *reported that *A. fumigatus *proteases promote host pulmonary inflammatory responses by downregulating PAR-2 expression through a TLR-4 dependent mechanism [58]. Thus, it is possible that proteases secreted by *A. fumigatus *growing on airway epithelium trigger IgG and IgE mediated allergic responses through crosstalk between PARs and TLRs-mediated signaling pathways leading to pulmonary complications as ABPA.

Bypassing of the tolerogenic mechanisms is also required to provoke Th2-mediated allergic responses in asthma patients [59]. Studies by Kheradmand *et al. *showed that fungal proteases have the capability to abort airway tolerance and when instilled in airways can activate Th2-mediated allergic responses without requiring adjuvant priming [49]. DCs also have an important role in maintaining tolerance in lungs by production of IL-10, an immunoregulatory cytokine that induces development of TGF-β expressing CD4+ T-regulatory cells (Tregs) [60]. In a recent study, Kriendler *et al. *showed that CD4+ T-cells from cohort of *A. fumigatus *colonized non-ABPA CF patients had an increased frequency of TGF-β-expressing Tregs compared to CF-ABPA patients [48]. This study suggested that tolerance against *A. fumigatus *antigens in CF-ABPA patients is defective and correlated to vitamin-D deficiency.

### Fungal Cell Wall components

The fungal cell wall is primarily composed of polysaccharides such as galactomannan, chitin, α- and β-glucans [61]. It is now well documented that the cell wall of swollen or germinated *A. fumigatus *conidia is composed of β-glucan, which triggers Dectin-1 mediated inflammatory responses [62-64]. Dectin-1 activated DCs promote the differentiation of Th17 and Th1 cells *in vivo *and can also convert Tregs into Th17 cells [65,66]. The role of Dectin-1 in airway epithelial cells is not well defined; however, recent studies did show Dectin-1 surface expression after TLR-2 stimulation with Mycobacteria and fungal antigens [67,68]}. CF airway epithelial cells were reported to have decreased expression of TLR-4 compared to healthy subjects leading to reduced innate immune responses to *P. aeruginosa *infection [69]. It is likely that the host genetic makeup determines TLR- and C-type lectin receptor(s)-specific immune responses to *A. fumigatus *cell wall components.

Chitin has been shown to induce host-chitinases in an *A. fumigatus*-infected guinea pig model which was diminished by an anti-fungal treatment [70]. Mice challenged with chitin demonstrated infiltration of IL-4 expressing eosinophils and basophils in lungs; this did not occur with chitin pretreated with acidic mammalian chitinase (AMCase) or in mice overexpressing AMCase [71]. AMCase is known to be expressed by murine airway epithelial cells and alveolar macrophages, and has been reported to impart anti-fungal immunity against chitin-containing organisms [72]. In this regard, Chen *et al. *recently reported *in vitro *inhibition of fungal activity by AMCase [73]. Thus, pulmonary immune response to various fungal components could determine the outcome towards protective or pathogenic.

### Host Genetic Susceptibility

#### Human leukocyte antigen (HLA) alleles

Genetic studies have revealed that the expression of specific MHC II alleles could determine development or protection against ABPA [74]. The frequency of HLA-DR2 (DRB1 *1501 and DRB1 *1503) or DR5 alleles has been reported to be higher in ABPA patients compared to CF or in asthmatic patients without ABPA [74]. This group also suggested the role of HLA-DRB1 *1502 as a resistance allele against the development of ABPA. Using humanized transgenic mice, they reported that *A. fumigatus *infection in DRB1*1501 and DRB1 *1503 strains cause profound ABPA-like pathology while the HLA-DRB1 *1502 strain mounts a protective Th1-type response [75]. It is now becoming clear that T cell receptor-MHC peptide ligand interactions play an important role in regulating the activation of immune responses and Th1/Th2 cytokine balance [76].

### Surfactant protein-A (SP-A) gene and mannan-binding lectin (MBL) polymorphisms

Genetic association studies have shown that polymorphisms in the SP-A and MBL gene lead to a predisposition to develop ABPA [77-79]. Saxena *et al. *showed that ABPA patients have a higher frequency of the A1660G SP-A2 allele than matched controls [77]. In line with this, another study also reported that ABPA patients have increased frequency of the T allele at T1492C and the G allele at G1649C of SP-A2 gene, and also higher frequency of TT genotype (71%) at 1492 of SP-A2 than controls [79]. Patients with the 1011A MBL allele were observed to have clinical features consistent with ABPA, such as increased eosinophilia, total IgE antibodies and lower FEV1 values [78]. Using murine models of allergic and invasive aspergillosis, the therapeutic potential of SP-A/D and MBL has been reported by Madan and colleagues (reviewed in [80]). These studies suggest a role of surfactant proteins and lectins as possible modulators of *A. fumigatus*-induced inflammation and allergy.

### Cytokine gene polymorphisms

Patients with ABPA have a higher frequency of the IL-15 +13689*A allele and A/A genotype with a lower frequency of the TNF-alpha-308*A/A genotype [81]. Another study reported that ABPA patients have a single nucleotide polymorphism (SNP) in the extracellular IL-4 receptor alpha, ile75val, which could lead to increased sensitivity to IL-4 stimulation [82]. Increased risk of *A. fumigatus *colonization in CF patients has been associated with polymorphisms in the promoter region of the IL-10 gene; there is a significant correlation between the -1082GG genotype with *A. fumigatus *colonization and ABPA [83].

### Polymorphisms in Chitinase and Chitinase-like proteins

Chitinases are enzymes known to cleave chitin present in fungal walls, parasites, insects and crustaceans [84]. Polymorphisms in two mammalian chitinases *viz. *AMCase and chitotrisidase (*CHIT*), and chitinase-like proteins such as YKL-40 have been reported to play important role in asthma susceptibility [84]. Polymorphisms in the AMCase gene are known to be associated with asthma [85,86]. Mutations in *CHIT1 *gene were also reported in patients with SAFS and can also be a risk factor for ABPA [87]. It has also been shown that high mold exposure can significantly modulate the effect of SNPs in *CHIT1 *gene on severe asthma exacerbations leading to increased hospitalizations- an example of gene-environment interactions as a determinant for an outcome of the disease [88].

## Conclusions

Taken together, it is now becoming evident that respiratory complications caused by *A. fumigatus *are the result of genes and the environment such that poor airway clearance of fungal spores drives a skewed adaptive response, and subsequent inflammation-driven lung damage. It is intriguing that we all inhale *A. fumigatus *conidia but only some people develop pathological responses to this fungus. Differences in make-up of multiple PRRs and cytokine genes in the propagation of inflammatory responses are involved in overall risks for allergic responses to fungi. More studies are needed to define precise interaction and decode genetic susceptibilities.

## List of abbreviations used

ABPA: Allergic bronchopulmonary aspergillosis; CF: Cystic fibrosis; Th: T-helper; DCs: Dendritic cells; PARs: Protease activated receptors; TLRs: Toll like receptors; CFTR: Cystic fibrosis transmembrane conductance regulator; SNP: Single nucleotide polymorphism; AMCase: Acidic mammalian chitinase; SAFS: Severe asthma with fungal sensitization; Tregs: T-regulatory cells; PRRs: Pattern recognition receptors; CHIT: Chitotrisidase.

## Competing interests

The authors declare that they have no competing interests.

## Authors' contributions

KM and NC wrote the manuscript. All authors read and approved the final manuscript.
